# Noninvasive pressure measurement with 4D phase contrast MRI in patients with aortic coarctations

**DOI:** 10.1186/1532-429X-14-S1-P106

**Published:** 2012-02-01

**Authors:** Elizabeth J Nett, Jie C Nguyen, Kevin Johnson, Oliver Wieben, Christopher Francois

**Affiliations:** 1 Medical Physics, University of Wisconsin, Madison, WI, USA; 2 Radiology, University of Wisconsin, Madison, WI, USA

## Summary

In this study, pressure gradients measured with 4D phase contrast MRI were compared to those measured with Doppler ultrasound in patients with aortic coarctations. We found good agreement between these methods as well as good correlation between pressure measurements and degree of stenosis.

## Background

**Figure 1:**
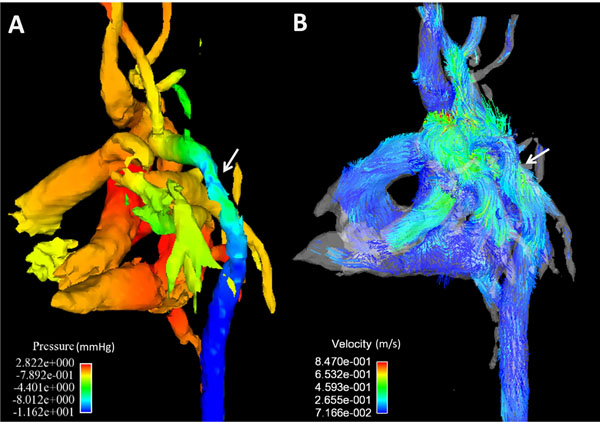
Pressure (A) and velocity maps (B) measured in a nine week old patient with an AoC using PC VIPR. The location of the coarctation is indicated by the white arrow.

Aortic coarctation (CoA) is defined as a congenital narrowing of the descending thoracic aorta severe enough to create pressure gradient. In clinical routine, invasive catheter pressure measurements are considered the gold standard. Pressure gradients can also be estimated noninvasively from Doppler ultrasound (US) or 2D phase contrast (PC) MRI using a simplified Bernoulli equation [1]. However, thoracic US measurements are not always possible, results can be user dependent, and do not provide information regarding temporal and spatial variations. 4D PC MRI with three-directional velocity encoding can be used to measure the spatial and temporal distribution of pressure gradients [2] as well as other hemodynamic parameters (Fig. 1). The purpose of this study was to compare pressure measurements made with US to those made with a radially undersampled 4D PC-MRI sequence in patients with coarctations.

## Methods

Seven subjects (2F/5M, mean age 22.7 years) with CoA were enrolled. Three patients were imaged before repair and four after. All patients had routine clinical transthoracic US exams.

### MRI

All patients were scanned on clinical 1.5T or 3T systems. Volumetric, time-resolved PC MRI data with 3-directional velocity encoding were acquired with dual-echo PC VIPR [3] and respiratory and retrospective cardiac gating: 1.25mm3 isotropic resolution, BW=125 kHz, TR 6.2ms, volume: 32cm x 32 cm x 20 cm, 12,000 dual echoes, scan time= ~13 min, Venc = 160 cm/s.

PC VIPR pressure gradients were derived using an iterative method based on the Navier-Stokes equation [2]. 3D visualization was achieved using EnSight (CEI). Quantification of pressure gradients was done using a Matlab (Mathworks) analysis tool. The PC VIPR and US pressure measurements were compared for statistical significant differences using the paired t-test (p<0.05).

## Results

A significant correlation between PC VIPR and US pressure measurements was observed (r = 0.84, P = 0.13). Overall, PC VIPR underestimated pressure differences compared with US . PC VIPR pressure measurements were compared with the grade of the stenosis and these measurements were also found to be correlated (r = 0.51, P = 0.14).

## Conclusions

This study demonstrates the utility of 4D PC MRI for measurement of 4D pressure gradients in aortic coarctations as compared with Doppler ultrasound as well as percent stenosis. Similar to the findings of a recent 4D MR flow study with Cartesian encoding, the peak pressure differences were lower than assessed with US [4]. 4D PC MRI can also be used to measure other important hemodynamic parameters such as WSS and OSI which have been linked to aneurysm formation. These noninvasive measures can possibly assist in the diagnosis and follow-up of patients with CHD.

## Funding

NIH NHLBI R01HL072260-05A1.

**Table 1:** Peak pressure differences measured across the aortic coarctation and with PC VIPR and Doppler ultrasound and associated percent stenosis for each patient

	Time Between Ultrasound and MRI	PC VIPR Pressure (mmHg)	Ultrasound Pressure (mmHg)	Stenosis (%)
**No Repair**	7 days	16.3	42**	32.8
	5 months	32.3	33.8	26.2
	6 days	16.3	23.4	18.4

**Post Repair**	17 months	5.5	0*	11.1
	1 month	6.6	0*	6.9
	3 months	26.6	35.1	30.1
	2 years	-1.6	0*	24.8

*Measurements not done because aorta measurements were within a normal range **Ultrasound report stated this measurement likely over-estimates pressure
